# Running rescues a fear-based contextual discrimination deficit in aged mice

**DOI:** 10.3389/fnsys.2015.00114

**Published:** 2015-08-11

**Authors:** Melody V. Wu, Victor M. Luna, René Hen

**Affiliations:** ^1^Department of Psychiatry, Columbia UniversityNew York, NY, USA; ^2^Division of Integrative Neuroscience, New York State Psychiatric InstituteNew York, NY, USA; ^3^Departments of Neuroscience and Pharmacology, Columbia UniversityNew York, NY, USA

**Keywords:** aging, exercise, pattern separation, dentate gyrus, neurogenesis

## Abstract

Normal aging and exercise exert extensive, often opposing, effects on the dentate gyrus (DG) of the hippocampus altering volume, synaptic function, and behaviors. The DG is especially important for behaviors requiring pattern separation—a cognitive process that enables animals to differentiate between highly similar contextual experiences. To determine how age and exercise modulate pattern separation in an aversive setting, young, aged, and aged mice provided with a running wheel were assayed on a fear-based contextual discrimination task. Aged mice showed a profound impairment in contextual discrimination compared to young animals. Voluntary exercise rescued this deficit to such an extent that behavioral pattern separation of aged-run mice was now similar to young animals. Running also resulted in a significant increase in the number of immature neurons with tertiary dendrites in aged mice. Despite this, neurogenesis levels in aged-run mice were still considerably lower than in young animals. Thus, mechanisms other than DG neurogenesis likely play significant roles in improving behavioral pattern separation elicited by exercise in aged animals.

## Introduction

Normal aging has been associated with impairments in a number of hippocampus-mediated spatial learning and memory tasks such as the Morris water and radial arm mazes (Rosenzweig and Barnes, 2003; O'Callaghan et al., 2009; Gazova et al., 2013), as well as decreased DG volume (Small et al., 2004), deficits in long-term potentiation induction, increased susceptibility to long-term depression, and reductions in DG synaptic contacts (Rosenzweig and Barnes, 2003; Gruart et al., 2008). Many of these hippocampal modifications are changed in the opposite direction following either voluntary or forced physical exercise. Running has been shown to improve performance in spatial discrimination tasks such as the Morris water maze and touch screen tests (Creer et al., 2010; Vivar et al., 2013; Voss et al., 2013) and associative tasks such as operant conditioning (e.g., lever pressing; Madroñal et al., 2010) and contextual fear conditioning (Clemenson et al., 2015) in adult mice. Not surprisingly, physical exercise has been proven to actually reverse age-related deficits in many behavioral, anatomical, and cellular domains (Colcombe and Kramer, 2003; van Praag et al., 2005; Albeck et al., 2006; Madroñal et al., 2010; Intlekofer and Cotman, 2013) though see also (Hansalik et al., 2006; Creer et al., 2010; Madroñal et al., 2010). The cognitive benefits of exercise may in fact supersede the normal aging process and may be used to alleviate some forms of memory decline in transgenic mouse models of Alzheimer's (e.g., water maze and operant conditioning, see García-Mesa et al., 2011) and in older human populations at risk for the disease (Larson et al., 2006; Lautenschlager et al., 2008).

A recent study has also shown that aging and exercise often exert opposing transcriptional regulation on the same genes in the hippocampus (Kohman et al., 2011). Several of these regulated genes are known to play a role in adult hippocampal neurogenesis. Adult neurogenesis in the subgranular zone of the DG is remarkably decreased in aged animals (Kuhn et al., 1996; Bondolfi et al., 2004) and this decrease can be partially reversed following exercise (van Praag et al., 2005; Kronenberg et al., 2006). However, though adult neurogenesis has been shown to be a necessary mechanism for the improvement of fine-scale spatial and contextual discrimination in adult mice (Clelland et al., 2009; Creer et al., 2010; Sahay et al., 2011a; Tronel et al., 2012), it may not fully account for the positive cognitive effects of exercise in young adult and aged animals (Meshi et al., 2006; Creer et al., 2010; Madroñal et al., 2010).

We thus examined the contribution of age and exercise to performance in a fear-based contextual discrimination task. The involvement of the DG in these types of associative learning has been well documented (Aimone et al., 2011; Sahay et al., 2011b) and recently highlighted by a study showing the differential participation of the DG in the encoding of contextual and cue signals (Carretero-Guillén et al., 2015). Performance on spatial and contextual discrimination tasks has previously been shown to correlate with levels of adult neurogenesis in the DG (Clelland et al., 2009; Creer et al., 2010; Sahay et al., 2011a; Tronel et al., 2012). These tasks have been proposed to require pattern separation, the process in which experiences are represented as distinct neural ensembles in the DG, thereby enabling animals to differentiate seemingly similar contexts from one another. Thus, testing for behavioral pattern separation in DG-dependent tasks could be useful in identifying endophenotypes related to overgeneralization of memory, which is observed in age-dependent and -independent cognitive decline as well as in anxiety related disorders (Yassa et al., 2011; Kheirbek et al., 2012).

We hypothesized that performance on a contextual fear discrimination task would be severely impaired in aged animals and rescued by voluntary exercise partly through neurogenesis-dependent mechanisms. While previous studies have examined aged rodents (Creer et al., 2010; Gracian et al., 2013) and humans (Toner et al., 2009; Stark et al., 2010) in reward-based and neutral contextual discrimination behaviors, our findings represent the first demonstration of the ameliorative effects of exercise on aging-related deficits in an aversion-based pattern separation task.

## Materials and methods

### Animals

Two and seventeen month old C57/Bl6 male mice were purchased from Charles River Laboratory and the NIA Aged Rodent Colony housed at Charles River, respectively. Mice were housed 3–4 to a cage, maintained on a 12:12 light cycle, and given food and water *ad libitum*. One week after arrival, half the aged animals were assigned to the aged-run group and provided with a running disc atop a polycarbonate igloo (Bio-Serv, NJ) and a cotton nestlet. Running distance was assessed via standard bike speedometers attached to the running discs. Visual observation confirmed that all animals in each cage chose to run on the wheel. Aged-run animals traveled an average of 1.3 km/day. Starting 1 week later, all animals were injected daily with phosphate buffered saline for the entirety of the experiment. Three i.p. injections of 100 mg/kg 5-bromo-2'-deoxyuridine (BrdU, Roche; Basel, Switzerland) dissolved 10 mg/mL in normal saline were administered to all animals 24 h apart beginning the first day of PBS administration. All procedures were approved by and complied with NIH AALAC and NYSPI and CU IACUC guidelines.

### Behavioral testing

**Figure 1:**
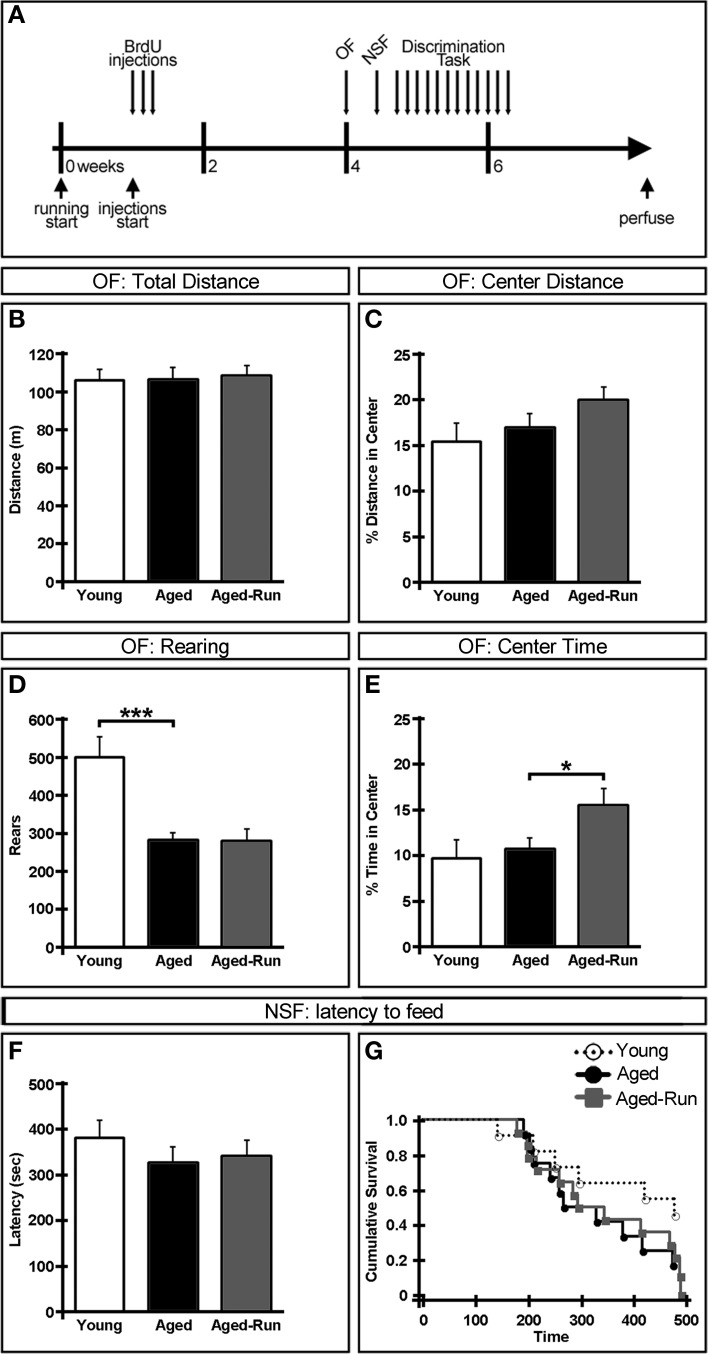
**Effect of age and exercise on anxiety-related behaviors. (A)** Experimental timeline. Daily PBS injections were initiated 1 week after aged-run animals were given access to a running disc. After 3 weeks of injections, animals were assessed in the open field (OF), novelty suppressed feeding (NSF), and discrimination task assays. **(B–E)** OF test. No difference was seen between animals in either total distance traveled **(B)** or percent center distance **(C)**. Young animals reared significantly more than aged mice **(D)**. Aged-run animals spent more time in the center of the arena compared to aged controls **(E)**. **(F,G)** NSF test. No differences were seen between any groups in latency to feed. Mean ± SEM. *n* = 11(young); *n* = 12(aged); *n* = 14(aged-run). ^*^p < 0.05, ^***^p < 0.001.

Assays were carried out during the light cycle with at least 48 h between tests (Figure 1A). To minimize stress associated with transportation, animals were wheeled into testing rooms at least 20 min prior to all assays. All assays were performed at least 1 h prior to daily injections.

### Anxiety-like behaviors

Open field (OF) and novelty suppressed feeding (NSF) assays were performed as previously described (Wu and Hen, 2014) in a dark (OF) or dimly lit (NSF) testing room. Briefly, OF assays were performed in a 43 × 43 cm plexiglass enclosed arena and ambulatory activity was automatically detected by infrared photobeam crosses over a 30 min period (Med-Associates; St Albans, VT). NSF was carried out in a plastic box (45 × 30 × 15 cm) covered with approximately 2 cm of sawdust bedding. Prior to the test, animals were food deprived for 24 h. A single pellet of standard chow was affixed to a raised platform covered with white filter paper. The mouse was placed in a corner of the arena and the latency to feed (defined as the mouse sitting on its haunches and biting the pellet with the use of its forepaws) was recorded (with a ceiling of 10 min).

### Contextual fear conditioning

Testing was performed in one side of a shuttle box (20.3 × 15.9 × 21.3 cm, Med-Associates; St Albans, VT) with two clear plexiglass walls, two aluminum walls, and a stainless steel grid floor, which was encased in a sound-dampening cubicle, as previously described (Sahay et al., 2011a). For training Context A, experimental room lights were on, the testing chamber was lit with a house light, ventilation was provided with a house fan, a mild anise scent was used as an olfactory cue, a non-alcoholic antiseptic was used to clean the chamber and grid floor between runs, and animals were transported from their home cages to the chambers and back using standard cages (Table 1). Mice were placed in the context for 180 s, received a single 2 s foot shock of 0.75 mA, and then removed from the context 15 s after the shock ended. For novel Context C, the experimental room was lit only with red light, the house light and fan were off, two foam inserts were used to cover the walls in an oblong fashion, the grid floor was covered with a plastic insert and a layer of corncob bedding, a mild mint scent was used as an olfactory cue, mild soap was used to clean the inserts between runs, and animals were transported using pie-shaped mouse cages (Table 1). Mice were placed in the context for 180 s and removed, with no shock delivery. All animals were trained in Context A on day 0, tested in Context A on day 1 with no shock presentation, and tested in Context C on day 2.

**Table 1:** **Contextual fear conditioning and contextual discrimination assay parameters**.

**Parameter**	**Context A**	**Context B**	**Context C**
Room lighting	On	Dim	Red
Chamber lighting	On	Off	Off
Ventilation	On	Off	Off
Olfactory cue	Anise	Lemon	Mint
Flooring	Steel grid bars	Steel grid bars	Plastic insert with layer of corncob bedding
Chamber wall	No additional walls	Plastic inserts to form circular walls	Foam inserts to form oblong walls
Cleanser	Non-alcoholic antiseptic	70% ethanol	Mild soap
Transport	Rectangle cages	Cardboard bucket	Pie cages

### Contextual discrimination

Beginning on day 3, animals were tested daily in Context A (the same training context as for contextual fear conditioning) with foot shock, returned to their home cage, and tested in Context B 1 h later (Sahay et al., 2011a). For similar Context B, experimental room lights were dimmed, the house light and fan were turned off, two plastic inserts were used to cover the walls in a circular fashion, a mild lemon scent was used as an olfactory cue, 70% ethanol was used to clean the inserts and grid floor between runs, and animals were transported using cardboard buckets (Table 1). Mice were placed in the context for 180 s and removed, with no shock delivery. Percent time freezing in each context was measured to calculate a discrimination ratio: (Freezing_ContextA_ – Freezing_ContextB_)/(Freezing_ContextA_ + Freezing_ContextB_). A discrimination ratio of 0 denotes equivalent freezing in the two contexts, indicating a lack of discrimination. Area under the curve was calculated by integrating the discrimination ratio over total days in the assay.

### Histology

Every sixth section was immunolabeled for either BrdU, Ki67, or DCX as previously described (Wu and Hen, 2014) 2 weeks subsequent to the conclusion of behavioral procedures. Primary antibodies included: rat anti-BrdU (1:200, Serotec OBT0030; Kidlington, UK), rabbit anti-Ki67 (1:100, Vector VP-RM04; Burlingame, CA), and goat anti-DCX (1:500, SCBT sc-8066; Dallas, TX). Secondary antibodies included: Cy3 donkey anti-rat (1:800, Jackson 712-165-150; West Grove, PA), AlexaFluor 488 anti-rabbit (1:300, Molecular Probes A21206; Eugene, OR), and biotinylated donkey anti-goat (1:500, Jackson 705-065-147). Sections were imaged on an AxioObserver A.1 or Axiovert 200 (Zeiss; Oberkochen, Germany). All BrdU, Ki67, and DCX immunolabeled cells spanning the DG were quantified and multiplied by six.

### Statistical analysis

**Figure 2:**
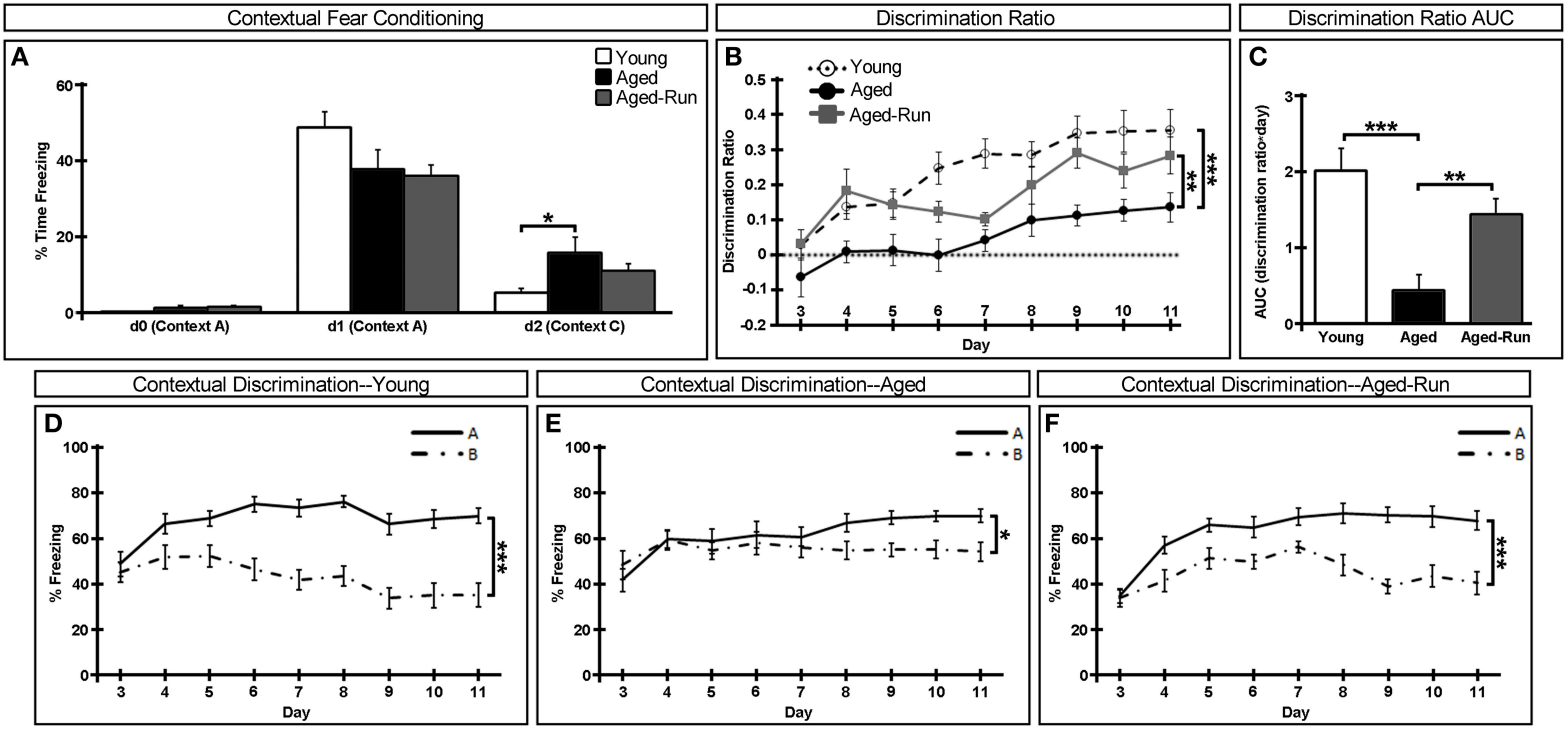
**Effect of age and exercise on a contextual discrimination task. (A)** There were no deficits in learning the one-shock contextual fear conditioning task in young, aged, aged-run mice. Animals in all groups froze upon re-exposure to the training one-shock context (Context A) on day 1 but displayed significantly lower levels of freezing upon exposure to a novel no-shock context (Context C) on day 2. **(B,C)** Aged animals were significantly impaired in the ability to discriminate between the training context (Context A) and a similar yet no-shock context (Context B) as assessed by the ratio of freezing in Context A compared to Context B, a deficit which is rescued in aged-run animals; AUC, area under the curve. **(D–F)** Percent time spent freezing in training Context A (solid line) or similar Context B (dotted line) over days 3 through 11 of the task. Mean ± SEM. *n* = 11(young); *n* = 12(aged); *n* = 14(aged-run). ^*^p < 0.05, ^**^p < 0.01, ^***^p < 0.001.

**Figure 3:**
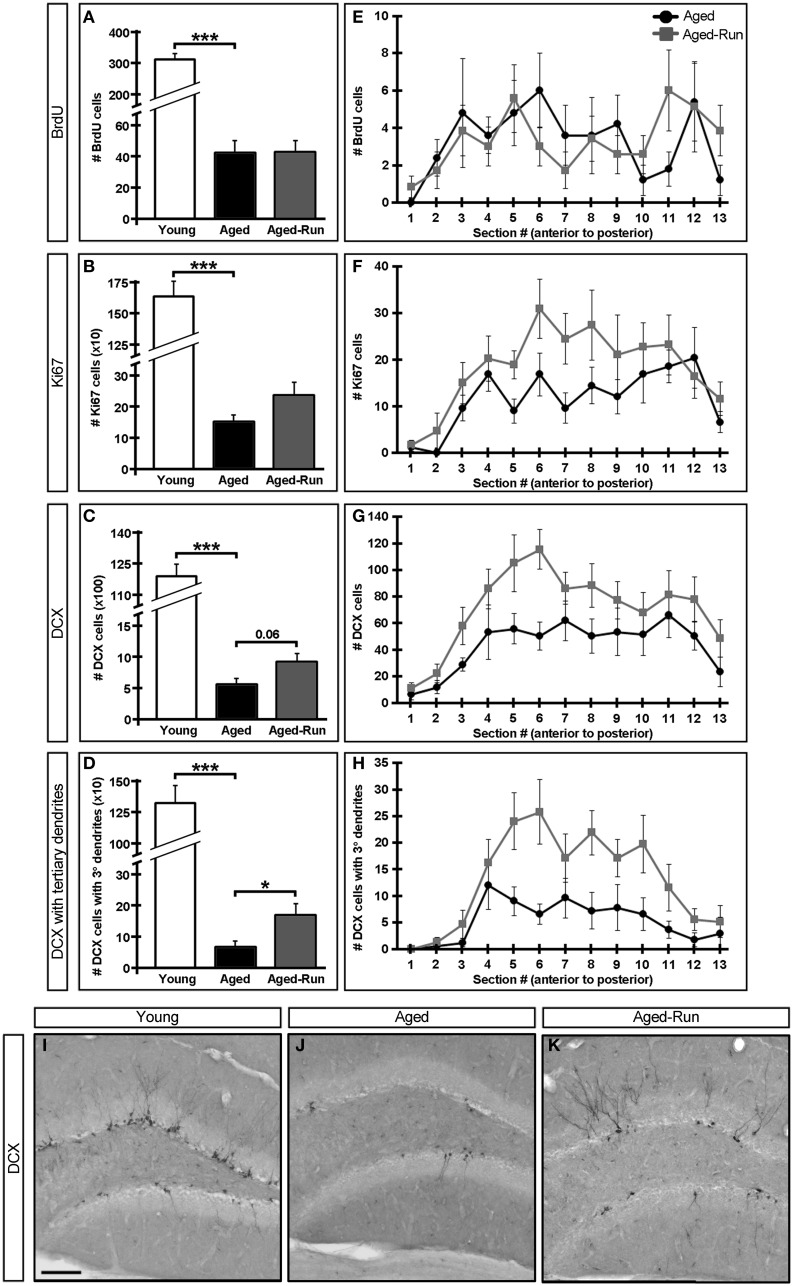
**Effect of age and exercise on neurogenesis. (A–D)** Quantification of neurogenesis in the DG of young, aged, and aged-run animals. Aged and aged-run mice have, on average, significantly fewer 7 week old BrdU+ cells **(A)**, Ki67+ cells **(B)**, DCX+ cells **(C)**, and DCX+ cells with tertiary or higher order dendrites **(D)** than young animals. Running increases some measures of neurogenesis in aged mice, but not to levels seen in young animals **(C,D)**. **(E–H)** Mean cell counts from aged and aged-run animals (summed in **A–D**) projected across the antero-posterior axis of the DG. Note the trend for DCX+ cell counts to be increased in aged-run compared to aged mice **(G)**. Aged-run mice had significantly more DCX+ cells with tertiary dendrites than aged animals **(H)**. **(I–K)** Representative coronal sections through the DG of young **(I)**, aged **(J)**, and aged-run **(K)** mice stained for doublecortin. Note changes both in number of DCX+ cells as well as complexity of such cells. Mean ± SEM. *n* = 10(young); *n* = 12(aged); *n* = 14(aged-run). ^*^p < 0.05, ^***^p < 0.001 **(A–H)**. Scale bar equals 100 μm **(I–K)**.

Data was analyzed by One-Way ANOVA between young and aged or between aged and aged-run groups for: OF total distance (Figure 1B), OF center distance (Figure 1C), OF rearing (Figure 1D), OF center time (Figure 1E), NSF latency to feed (Figure 1F), area under the curve of the contextual discrimination ratio (Figure 2C), and number of cells of each histological marker (Figures 3A–D). Homogeneity of variance was confirmed between each pair of two groups with the *F*-test. Data was analyzed by repeated measures ANOVA between days 0 and 1 or between days 1 and 2 for contextual fear conditioning in each group (Figure 2A). Data was analyzed by one-way repeated measures ANOVA between contexts A and B across days 3–11 for contextual discrimination in each group (Figures 2D–F) and between aged and aged-run groups across sections 1–13 for cell counts of each histological marker (Figures 3E–H). Data was analyzed by one-way repeated measures ANOVA between young and aged or between aged and aged-run groups across days 3–11 for contextual discrimination ratio (Figure 2B). Latency to feed in the NSF was also analyzed using Kaplan–Meier survival curves followed by Mantel-Cox log-rank tests (Figure 1G). *P* < 0.05 were deemed statistically significant. A full listing of statistical results is provided in Table 2.

**Table 2:** **Statistical analyses**.

**Behavior**	**Test**	**Independent variable**	**Factor**	**Fig**	***df***	**Stat**	**Sig**
OF: total distance	One-Way ANOVA	Young vs. aged	Group	**1B**	*F*_(1, 21)_	0.0004	0.99
		Aged vs. aged-run			*F*_(1, 24)_	0.06	0.81
OF: center distance	One-Way ANOVA	Young vs. aged	Group	**1C**	*F*_(1, 21)_	0.36	0.55
		Aged vs. aged-run			*F*_(1, 24)_	2.13	0.16
OF: rearing	One-Way ANOVA	Young vs. aged	Group	**1D**	*F*_(1, 21)_	15.57	0.0007
		Aged vs. aged-run			*F*_(1, 24)_	0.01	0.93
OF: center time	One-Way ANOVA	Young vs. aged	Group	**1E**	*F*_(1, 21)_	0.21	0.65
		Aged vs. aged-run			*F*_(1, 24)_	4.66	0.04
NSF: latency to feed	One-Way ANOVA	Young vs. aged	Group	**1F**	*F*_(1, 21)_	1.12	0.30
		Aged vs. aged-run			*F*_(1, 24)_	0.10	0.75
	Mantel-cox logrank	Young vs. aged vs. aged-run	Group	**1G**	$\chi_{2,\;N=37}^{2}$	2.38	0.30
Contextual fear conditioning: young	Repeated measures ANOVA	d0 vs. d1	Day	**2A**	*F*_(1, 10)_	127.87	< 0.0001
		d1 vs. d2			*F*_(1, 10)_	89.33	< 0.0001
Contextual fear conditioning: aged	Repeated measures ANOVA	d0 vs. d1	Day	**2A**	*F*_(1, 11)_	47.54	< 0.0001
		d1 vs. d2			*F*_(1, 11)_	25.10	0.003
Contextual fear conditioning: aged-run	Repeated measures ANOVA	d0 vs. d1	Day	**2A**	*F*_(1, 13)_	141.41	< 0.0001
		d1 vs. d2			*F*_(1, 13)_	85.60	< 0.0001
Contextual discrimination ratio		Young vs. aged	Group		*F*_(1, 21)_	19.09	0.0003
			Day		*F*_(8, 168)_	15.70	< 0.0001
			Group × day		*F*_(8, 168)_	1.62	0.12
	One-Way repeated measures ANOVA	Aged vs. aged-run	Group	**2B**	*F*_(1, 24)_	12.43	0.002
			Day		*F*_(8, 192)_	7.92	< 0.0001
			Group × day		*F*_(8, 192)_	0.50	0.85
		Young vs. aged vs. aged-run	Group		*F*_(2, 34)_	10.82	0.0002
			Day		*F*_(8, 272)_	16.93	< 0.0001
			Group × day		*F*_(16, 272)_	1.27	0.22
Contextual discrimination ratio AUC	One-Way ANOVA	Young vs. aged	Group	**2C**	*F*_(1, 21)_	19.84	0.0002
		Aged vs. aged-run			*F*_(1, 24)_	11.79	0.002
Contextual discrimination across days: young	One-Way repeated measures ANOVA	Context A vs. B	Context	**2D**	*F*_(1, 10)_	65.76	< 0.0001
			Day		*F*_(8, 80)_	7.53	< 0.0001
			Context × day		*F*_(8, 80)_	11.90	< 0.0001
Contextual discrimination across days: aged	One-Way repeated measures ANOVA	Context A vs. B	Context	**2E**	*F*_(1, 11)_	7.14	0.02
			Day		*F*_(8, 88)_	2.98	0.005
			Context × day		*F*_(8, 88)_	4.76	< 0.0001
Contextual discrimination across days: aged-run	One-Way repeated measures ANOVA	Context A vs. B	Context	**2F**	*F*_(1, 13)_	59.38	< 0.0001
			Day		*F*_(8, 104)_	13.63	< 0.0001
			Context × day		*F*_(8, 104)_	6.96	< 0.0001
Number of cells: BrdU	One-Way ANOVA	Young vs. aged	Group	**3A**	*F*_(1, 19)_	141.01	< 0.0001
		Aged vs. aged-run			*F*_(1, 22)_	0.004	0.95
Number of cells: Ki67	One-Way ANOVA	Young vs. aged	Group	**3B**	*F*_(1, 19)_	132.36	< 0.0001
		Aged vs. aged-run			*F*_(1, 22)_	2.52	0.13
Number of cells: DCX	One-Way ANOVA	Young vs. aged	Group	**3C**	*F*_(1, 19)_	410.17	< 0.0001
		Aged vs. aged-run			*F*_(1, 22)_	3.95	0.06
Number of cells: DCX with tertiary dendrites	One-Way ANOVA	Young vs. aged	Group	**3D**	*F*_(1, 19)_	72.83	< 0.0001
		Aged vs. aged-run			*F*_(1, 22)_	5.21	0.03
Number of cells across sections: BrdU	One-Way repeated measures ANOVA	Aged vs. aged-run	Group	**3E**	*F*_(1, 22)_	0.004	0.95
			Section		*F*_(12, 264)_	1.81	0.05
			Group × section		*F*_(12, 264)_	0.88	0.57
Number of cells across sections: Ki67	One-Way repeated measures ANOVA	Aged vs. aged-run	Group	**3F**	*F*_(1, 22)_	2.52	0.13
			Section		*F*_(12, 264)_	5.51	< 0.0001
			Group × section		*F*_(12, 264)_	0.84	0.61
Number of cells across sections: DCX	One-Way repeated measures ANOVA	Aged vs. aged-run	Group	**3G**	*F*_(1, 22)_	3.95	0.06
			Section		*F*_(12, 264)_	8.97	< 0.0001
			Group × section		*F*_(12, 264)_	1.04	0.41
Number of cells across sections: DCX with tertiary dendrites	One-Way repeated measures ANOVA	Aged vs. aged-run	Group	**3H**	*F*_(1, 22)_	5.21	0.03
			Section		*F*_(12, 264)_	8.76	< 0.0001
			Group × section		*F*_(12, 264)_	2.18	0.01

Analyses were deemed significant if p-values were below 0.05. Behavior, specific behavioral measurement; Test, statistical test; Fig, figure; df, degrees of freedom; Stat, F- or χ^2^-statistic (as appropriate); Sig, p-value.

## Results

### Anxiety-related behaviors

To examine anxiety-related behaviors, young (3 month old), aged (18 month old), and aged-run animals (average travel of 1.3 km/day) were tested on the OF and NSF tests. These behaviors were assessed in order to rule out baseline anxiety differences between groups which could confound results from following cognitive tasks. All animals exhibited comparable levels of exploration and anxiety as measured by total distance traveled [Figure 1B; young vs. aged: *F*_(1, 21)_ = 3.60 × 10^−4^, *p* = 0.99; aged vs. aged-run: *F*_(1, 24)_ = 0.06, *p* = 0.81] and percent of distance traveled in the center of the arena [Figure 1C; young vs. aged: *F*_(1, 21)_ = 0.36, *p* = 0.55; aged vs. aged-run: *F*_(1, 24)_ = 2.13, *p* = 0.16]. However, young animals displayed significantly more rearing behavior than aged mice [Figure 1D; *F*_(1, 21)_ = 15.57, *p* = 0.0007] while running animals spent more time in the center of the arena compared to age-matched controls [Figure 1E; *F*_(1, 24)_ = 4.66, *p* = 0.04]. No differences were seen in the latency to begin feeding in the NSF test [Figures 1F,G; young vs. aged: *F*_(1, 21)_ = 1.12, *p* = 0.30; aged vs. aged-run: *F*_(1, 24)_ = 0.10, *p* = 0.75; $\chi_{2,\;N\left(37\right)}^{2}=2.38$, *p* = 0.30], suggesting that this anxiety-related behavior is independent of both age and exercise.

### Contextual conditioning and discrimination

To examine the cognitive abilities of young, aged, and aged-run mice, animals were tested in a one-shock contextual fear conditioning task (Figure 2A). All animals acquired conditioning to a foot shock in Context A, demonstrably freezing upon re-exposure to the context 24 h later [freezing on d0 vs. d1—young: *F*_(1, 10)_ = 127.87, *p* = 5.53 × 10^−10^; aged: *F*_(1, 11)_ = 47.54, *p* = 3.48 × 10^−7^; aged-run: *F*_(1, 13)_ = 141.41, *p* = 2.10 × 10^−11^]. Upon exposure to the novel no-shock Context C 24 h later, all animals displayed significantly lower levels of freezing, indicating that neither age nor exercise altered the ability of the animals to distinguish between decidedly different contexts [freezing on d1 vs. d2—young: *F*_(1, 10)_ = 89.33, *p* = 5.74 × 10^−9^; aged: *F*_(1, 11)_ = 25.10, *p* = 2.59 × 10^−3^; aged-run: *F*_(1, 13)_ = 85.60, *p* = 2.49 × 10^−7^]. It should be noted, however, that aged animals displayed significantly more freezing in the neutral Context C on day 2 compared to young animals [*F*_(1, 21)_ = 5.80, *p* = 0.03].

The ability of animals to differentiate between highly similar contexts was then tested in a contextual fear discrimination task. On the first day of this paradigm, all animals froze equivocally in Context A and in the similar yet no-shock Context B, indicating a generalization of contextual fear across all groups [day 3 of Figures 2D–F; young: *F*_(1, 10)_ = 0.90, *p* = 0.37; aged: *F*_(1, 11)_ = 2.20, *p* = 0.17; aged-run: *F*_(1, 13)_ = 0.06, *p* = 0.81]. However, over the course of the next 8 days, aged mice were shown to be significantly impaired in their ability to distinguish between the two similar contexts compared to young animals though aged animals do eventually learn to discriminate [Figure 2B; discrimination ratio: *F*_(1, 21)_ = 19.09, *p* = 0.0003; Figure 2C; area under the curve: *F*_(1, 21)_ = 19.84, *p* = 0.0002; Figure 2D; young discrimination: *F*_(1, 10)_ = 65.76, *p* < 0.0001; Figure 2E; aged discrimination: *F*_(1, 11)_ = 7.14, *p* = 0.02]. Interestingly, behavior in this aversive discrimination task was no longer impaired when aged animals were provided with a running wheel as exercising animals performed significantly better than their age-matched controls [Figure 2B; discrimination ratio: *F*_(1, 24)_ = 12.43, *p* = 0.002; Figure 2C area under the curve: *F*_(1, 24)_ = 11.79, *p* = 0.002; Figure 2F; aged-run discrimination: *F*_(1, 13)_ = 59.38, *p* < 0.0001].

### Adult hippocampal neurogenesis

**Figure 4:**
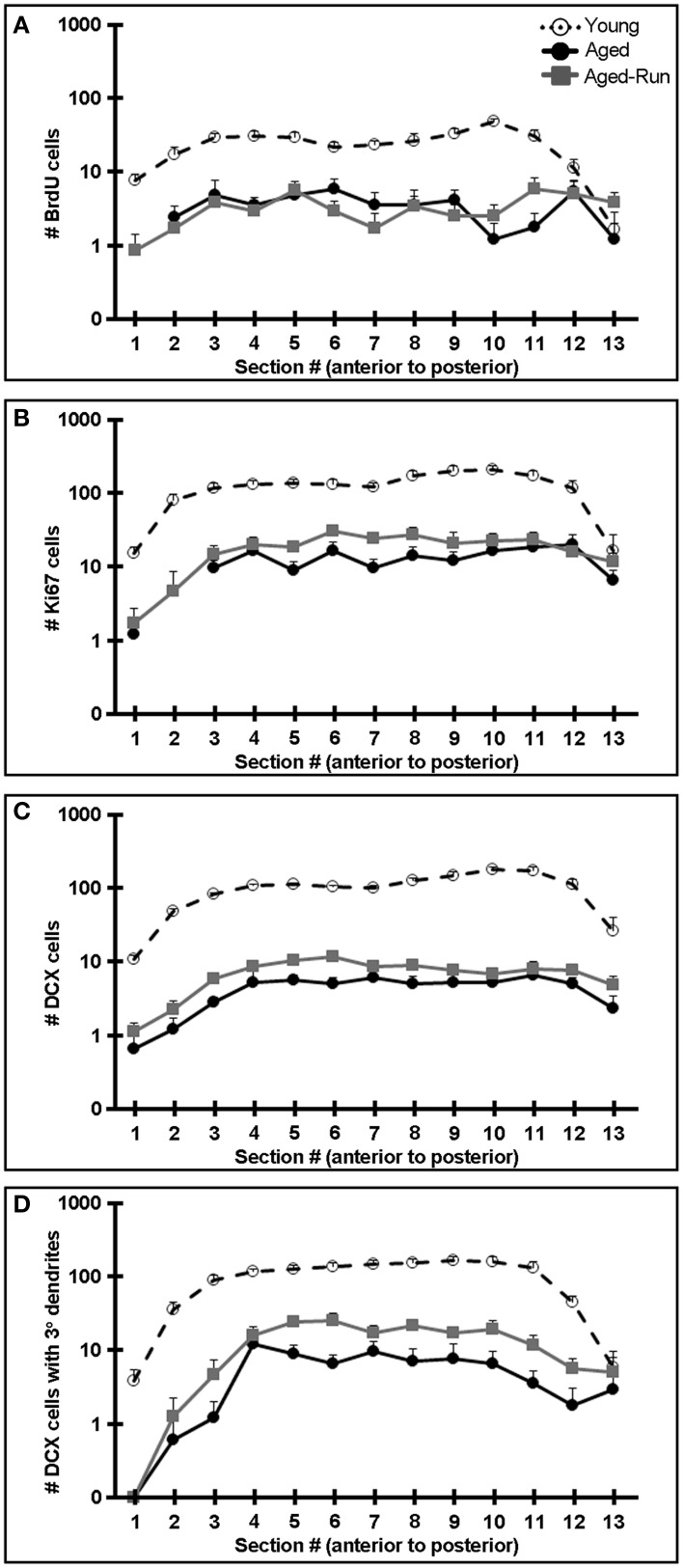
**Comparison of neurogenesis between young vs. aged and aged-run mice across the antero-posterior axis of the DG. (A–D)** The average number of 7 week old BrdU+ cells **(A)**, Ki67+ cells **(B)**, DCX+ cells **(C)**, and DCX+ cells with tertiary or higher order dendrites **(D)** in young, aged, and aged-run animals along the DG antero-posterior axis plotted on a logarithmic axis. Young mice had markedly higher levels of neurogenesis across all measures compared to aged and aged-run mice. Mean + SEM. *n* = 10(young); *n* = 12(aged); *n* = 14(aged-run).

Improved performance on the contextual discrimination task had previously been demonstrated to correlate with levels of adult hippocampal neurogenesis (Sahay et al., 2011a). We therefore examined markers of proliferation (Ki67+ cells) and survival (7 week old BrdU+ cells), as well as the numbers of immature neurons (DCX+ cells) and complexity of their dendrites (DCX+ cells with tertiary dendrites) (Wang et al., 2008) in the DG of young, aged, and aged-run animals. Young mice had significantly greater numbers of 7 week old BrdU+, Ki67+, and DCX+ cells compared to aged mice [Figures 3A–D,I,J; BrdU: *F*_(1, 19)_ = 141.01, *p* = 3.09 × 10^−10^; Ki67: *F*_(1, 19)_ = 132.36, *p* = 5.26 × 10^−10^; DCX: *F*_(1, 19)_ = 410.17, *p* = 2.53 × 10^−14^; DCX with tertiary dendrites: *F*_(1, 19)_ = 72.83, *p* = 6.33 × 10^−8^], a difference reflected across the antero-posterior axis of the dentate gyrus (Figure 4). Thus, running does not increase neurogenesis in aged mice to levels comparable to young animals despite aged-run mice displaying similar contextual discrimination abilities as young mice (Figures 2D,F).

While the numbers of BrdU+ and Ki67+ cells were indistinguishable between aged and aged-run animals [Figures 3A,B,E,F; BrdU: *F*_(1, 22)_ = 0.004, *p* = 0.95; Ki67: *F*_(1, 22)_ = 2.52, *p* = 0.13], there was a trend toward more DCX+ cells in running animals compared to age-matched controls [Figures 3C,G,J,K; *F*_(1, 22)_ = 3.95, *p* = 0.06]. Most notably, there were significantly more DCX+ cells with tertiary or higher order dendritic branching in aged-run animals compared to sedentary aged mice [Figures 3D,J,K; *F*_(1, 22)_ = 5.21, *p* = 0.03]. Increased numbers of DCX+ cells with tertiary or higher order dendritic branching were seen across the antero-posterior axis in aged-run animals [Figure 3H; *F*_(12, 264)_ = 2.183, *p* = 0.01].

## Discussion

With the average age of the world's population rapidly rising (United Nations World Population Ageing 2013), the need for studies investigating aging-related cognitive impairments has become increasingly important. A burgeoning number of studies have demonstrated that physical activity can reverse cognitive decline observed in normal aging in both humans and rodents (Hillman et al., 2008; Vivar et al., 2013). For instance, cerebral blood volume in the DG is increased following exercise in middle-aged humans (Pereira et al., 2007) and the dramatic drop in adult neurogenesis that accompanies aging can be improved following physical exercise in mice (van Praag et al., 2005).

Here we find that, in comparison to young mice, aged animals were impaired in a DG-dependent aversive pattern separation task in which they were required to discriminate between highly similar contexts. However, given the opportunity to exercise voluntarily, aged animals performed significantly better than age-matched controls and nearly as well as young mice. In our paradigm, aged mice ran an average of 1.3 km/day. This distance is shorter than the 3.9 km/day (van Praag et al., 2005) and 5.4 km/day (Creer et al., 2010) previously reported by other groups. One possible explanation for this difference is that our animals were group housed while previous measurements were carried out on individually housed animals. The decision to group house animals was made to minimize social isolation stress (Kalliokoski et al., 2014). We note, however, that even with this shorter running distance, aged-run mice displayed massive cognitive improvement compared to control mice of the same age. It would appear that increased neurogenesis in the DG can partially account for this improvement since the number of immature neurons, especially those with tertiary dendrites, was increased in aged-run mice compared to aged controls consistent with previous studies (Bednarczyk et al., 2009; Tanti and Belzung, 2013).

However, it is likely that the increased number of immature neurons alone is not responsible for the improved performance in the fear-based pattern separation task. Normal aging and voluntary exercise exert global cellular and transcriptional changes (Kohman et al., 2011) with widespread downstream effects. It will therefore be important to directly manipulate adult-born neurons, via targeted ablation and genetic manipulation (Wong-Goodrich et al., 2010; Sahay et al., 2011a), to determine how neurogenesis impacts contextual fear discrimination in aged and aged-run mice.

We note that our findings do not agree with previous work showing that voluntary exercise does not rescue spatial pattern separation in aged mice (Creer et al., 2010) likely because our fear-based task evokes disparate neural networks than those utilized in their appetite-based spatial paradigm (Hayes et al., 2014). It is also possible that performance in our fear-based task is more amenable to rescue due to a negative-valence bias in memory strength (Mickley Steinmetz et al., 2012). Indeed, aged animals were unimpaired in their ability to differentiate between dissimilar contexts (a low demand task) despite performing poorly when contexts were highly similar (a high demand task). In contrast, aged mice were impaired in learning both high and low demand versions of a reward-based spatial discrimination task (Creer et al., 2010). These results highlight that running does not globally improve learning (for instance, classical eye-blink conditioning is unaffected by exercise; Madroñal et al., 2010) and that its positive effects may be most beneficial for demanding cognitive behaviors that utilize specific sets of neural networks and computational processes like pattern separation. This is especially important when determining what cognitive behaviors could actually be alleviated in aging-related diseases like Alzheimer's (Larson et al., 2006; Lautenschlager et al., 2008; García-Mesa et al., 2011).

Similar to Creer et al. (2010); Madroñal et al. (2010), but unlike van Praag et al. (2005), we do not see an increase in the number of proliferating or 7 week-old cells in the DG of aged mice following voluntary exercise, supporting the view that neurogenesis appears to be related to running primarily in young animals (Creer et al., 2010; Madroñal et al., 2010; Vivar et al., 2013; Voss et al., 2013). We cannot, of course, exclude the possibility that we have underestimated the number of new neurons due to insufficient BrdU incorporation (Taupin, 2007) during our 3 day BrdU administration protocol as opposed to five (Creer et al., 2010) or seven (van Praag et al., 2005) days of BrdU injections. However, we do see a slight increase in the number of neurons expressing the immature cell marker doublecortin. That being said, previous reports have shown that adult neurogenesis alone may not fully account for the ameliorative effects of enriched environments and exercise on hippocampus-dependent behaviors (Meshi et al., 2006; Madroñal et al., 2010).

To determine if neurogenesis does contribute to exercise-induced rescue of behavioral pattern separation in older animals, future studies will need to assess if ablation of neurogenesis (e.g., via x-irradiation) in aged-run mice hinders exercise-induced improvement of contextual fear discrimination. Nonetheless, the robust amelioration of behavioral pattern separation elicited by exercise in aged mice suggests the possibility that environmental or pharmacological manipulations that relate to neurogenesis may be effective in reversing specific aspects of cognitive decline observed during the course of aging.

### Conflict of interest statement

Melody V. Wu and Victor M. Luna declare no competing financial interests. René Hen receives compensation as a consultant for Lundbeck, Roche, and Servier.

## References

[B1] Aimone J. B. Deng W. Gage F. H. (2011). Resolving new memories: a critical look at the dentate gyrus, adult neurogenesis, and pattern separation. Neuron 70, 589–596. 10.1016/j.neuron.2011.05.010 21609818 PMC3240575

[B2] Albeck D. S. Sano K. Prewitt G. E. Dalton L. (2006). Mild forced treadmill exercise enhances spatial learning in the aged rat. Behav. Brain Res. 168, 345–348. 10.1016/j.bbr.2005.11.008 16388860

[B3] Bednarczyk M. R. Aumont A. Décary S. Bergeron R. Fernandes K. J. (2009). Prolonged voluntary wheel-running stimulates neural precursors in the hippocampus and forebrain of adult CD1 mice. Hippocampus 19, 913–927. 10.1002/hipo.20621 19405143

[B4] Bondolfi L. Ermini F. Long J. M. Ingram D. K. Jucker M. (2004). Impact of age and caloric restriction on neurogenesis in the dentate gyrus of C57BL/6 mice. Neurobiol. Aging 25, 333–340. 10.1016/S0197-4580(03)00083-6 15123339

[B5] Carretero-Guillen A. Pacheco-Calderon R. Delgado-Garcia J. M. Gruart A. (2015). Involvement of hippocampal inputs and intrinsic circuit in the acquisition of context and cues during classical conditioning in behaving rabbits. Cereb. Cortex 25, 1278–1289. 10.1093/cercor/bht321 24243618

[B6] Clelland C. D. Choi M. Romberg C. Clemenson G. D. Jr. Fragniere A. Tyers P.. (2009). A functional role for adult hippocampal neurogenesis in spatial pattern separation. Science 325, 210–213. 10.1126/science.1173215 19590004 PMC2997634

[B7] Clemenson G. D. Lee S. W. Deng W. Barrera V. R. Iwamoto K. S. Fanselow M. S.. (2015). Enrichment rescues contextual discrimination deficit associated with immediate shock. Hippocampus 25, 385–392. 10.1002/hipo.22380 25330953 PMC4398310

[B8] Colcombe S. Kramer A. F. (2003). Fitness effects on the cognitive function of older adults: a meta-analytic study. Psychol. Sci. 14, 125–130. 10.1111/1467-9280.t01-1-01430 12661673

[B9] Creer D. J. Romberg C. Saksida L. M. van Praag H. Bussey T. J. (2010). Running enhances spatial pattern separation in mice. Proc. Natl. Acad. Sci. U.S.A. 107, 2367–2372. 10.1073/pnas.0911725107 20133882 PMC2836679

[B10] García-Mesa Y. López-Ramos J. C. Giménez-Llort L. Revilla S. Guerra R. Gruart A.. (2011). Physical exercise protexts against Alzheimer's disease in 3xTg-AD mice. J. Alzheimers. Dis. 24, 421–454. 10.3233/JAD-2011-101635 21297257

[B11] Gazova I. Laczó J. Rubinova E. Mokrisova I. Hyncicova E. Andel R.. (2013). Spatial navigation in young versus older adults. Front. Aging Neurosci. 5:94. 10.3389/fnagi.2013.00094 24391585 PMC3867661

[B12] Gracian E. I. Shelley L. E. Morris A. M. Gilbert P. E. (2013). Age-related changes in place learning for adjacent and separate locations. Neurobiol. Aging 34, 2304–2309. 10.1016/j.neurobiolaging.2013.03.033 23618871 PMC3706539

[B13] Gruart A. López-Ramos J. C. Munoz M. D. Delgado-García J. M. (2008). Aged wild-type and APP, PS1, and APP+PS1 mice present similar deficits in associative learning and synaptic plasticity independent of amyloid load. Neurobiol. Dis. 30, 439–450. 10.1016/j.nbd.2008.03.001 18442916

[B14] Hansalik M. Skalicky M. Viidik A. (2006). Impairment of water maze behaviour with ageing is counteracted by maze learning earlier in life but not by physical exercise, food restriction or housing conditions. Exp. Gerontol. 41, 169–174. 10.1016/j.exger.2005.11.002 16361075

[B15] Hayes D. J. Duncan N. W. Xu J. Northoff G. (2014). A comparison of neural responses to appetitive and aversive stimuli in humans and other mammals. Neurosci. Biobehav. Rev. 45C, 350–368. 10.1016/j.neubiorev.2014.06.018 25010558

[B16] Hillman C. H. Erickson K. I. Kramer A. F. (2008). Be smart, exercise your heart: exercise effects on brain and cognition. Nat. Rev. Neurosci. 9, 58–65. 10.1038/nrn2298 18094706

[B17] Intlekofer K. A. Cotman C. W. (2013). Exercise counteracts declining hippocampal function in aging and Alzheimer's disease. Neurobiol. Dis. 57, 47–55. 10.1016/j.nbd.2012.06.011 22750524

[B18] Kalliokoski O. Teilmann A. C. Jacobsen K. R. Abelson K. S. Hau J. (2014). The lonely mouse - single housing affects serotonergic signaling integrity measured by 8-OH-DPAT-induced hypothermia in male mice. PLoS ONE 9:e111065. 10.1371/journal.pone.0111065 25436462 PMC4249803

[B19] Kheirbek M. A. Klemenhagen K. C. Sahay A. Hen R. (2012). Neurogenesis and generalization: a new approach to stratify and treat anxiety disorders. Nat. Neurosci. 15, 1613–1620. 10.1038/nn.3262 23187693 PMC3638121

[B20] Kohman R. A. Rodriguez-Zas S. L. Southey B. R. Kelley K. W. Dantzer R. Rhodes J. S. (2011). Voluntary wheel running reverses age-induced changes in hippocampal gene expression. PLoS ONE 6:e22654. 10.1371/journal.pone.0022654 21857943 PMC3152565

[B21] Kronenberg G. Bick-Sander A. Bunk E. Wolf C. Ehninger D. Kempermann G. (2006). Physical exercise prevents age-related decline in precursor cell activity in the mouse dentate gyrus. Neurobiol. Aging 27, 1505–1513. 10.1016/j.neurobiolaging.2005.09.016 16271278

[B22] Kuhn H. G. Dickinson-Anson H. Gage F. H. (1996). Neurogenesis in the dentate gyrus of the adult rat: age-related decrease of neuronal progenitor proliferation. J. Neurosci. 16, 2027–2033. 8604047 10.1523/JNEUROSCI.16-06-02027.1996 PMC6578509

[B23] Larson E. B. Wang L. Bowen J. D. McCormick W. C. Teri L. Crane P.. (2006). Exercise is associated with reduced risk for incident dementia among persons 65 years of age and older. Ann. Intern. Med. 144, 73–81. 10.7326/0003-4819-144-2-200601170-00004 16418406

[B24] Lautenschlager N. T. Cox K. L. Flicker L. Foster J. K. van Bockxmeer F. M. Xiao J.. (2008). Effect of physical activity on cognitive function in older adults at risk for Alzheimer disease: a randomized trial. JAMA 300, 1027–1037. 10.1001/jama.300.9.1027 18768414

[B25] Madroñal N. Lopez-Aracil C. Rangel A. del Rio J. A. Delgado-Garcia J. M. Gruart A. (2010). Effects of enriched physical and social environment on motor performance, associative learning, and hippocampal neurogenesis in mice. PLoS ONE 5:e11130. 10.1371/journal.pone.0011130 20559565 PMC2886110

[B26] Meshi D. Drew M. R. Saxe M. Ansorge M. S. David D. Santarelli L.. (2006). Hippocampal neurogenesis is not required for behavioral effects of environmental enrichment. Nat. Neurosci. 9, 729–731. 10.1038/nn1696 16648847

[B27] Mickley Steinmetz K. R. Schmidt K. Zucker H. R. Kensinger E. A. (2012). The effect of emotional arousal and retention delay on subsequent-memory effects. Cogn. Neurosci. 3, 150–159. 10.1080/17588928.2012.677421 24171733 PMC3818726

[B28] O'Callaghan R. M. Griffin E. W. Kelly A. M. (2009). Long-term treadmill exposure protects against age-related neurodegenerative change in the rat hippocampus. Hippocampus 19, 1019–1029. 10.1002/hipo.20591 19309034

[B29] Pereira A. C. Huddleston D. E. Brickman A. M. Sosunov A. A. Hen R. McKhann G. M.. (2007). An *in vivo* correlate of exercise-induced neurogenesis in the adult dentate gyrus. Proc. Natl. Acad. Sci. U.S.A. 104, 5638–5643. 10.1073/pnas.0611721104 17374720 PMC1838482

[B30] Rosenzweig E. S. Barnes C. A. (2003). Impact of aging on hippocampal function: plasticity, network dynamics, and cognition. Prog. Neurobiol. 69, 143–179. 10.1016/S0301-0082(02)00126-0 12758108

[B31] Sahay A. Scobie K. N. Hill A. S. O'Carroll C. M. Kheirbek M. A. Burghardt N. S.. (2011a). Increasing adult hippocampal neurogenesis is sufficient to improve pattern separation. Nature 472, 466–470. 10.1038/nature09817 21460835 PMC3084370

[B32] Sahay A. Wilson D. A. Hen R. (2011b). Pattern separation: a common function for new neurons in hippocampus and olfactory bulb. Neuron 70, 582–588. 10.1016/j.neuron.2011.05.012 21609817 PMC3109085

[B33] Small S. A. Chawla M. K. Buonocore M. Rapp P. R. Barnes C. A. (2004). Imaging correlates of brain function in monkeys and rats isolates a hippocampal subregion differentially vulnerable to aging. Proc. Natl. Acad. Sci. U.S.A. 101, 7181–7186. 10.1073/pnas.0400285101 15118105 PMC406486

[B34] Stark S. M. Yassa M. A. Stark C. E. (2010). Individual differences in spatial pattern separation performance associated with healthy aging in humans. Learn. Mem. 17, 284–288. 10.1101/lm.1768110 20495062 PMC2884287

[B35] Tanti A. Belzung C. (2013). Neurogenesis along the septo-temporal axis of the hippocampus: are depression and the action of antidepressants region-specific? Neuroscience 252, 234–252. 10.1016/j.neuroscience.2013.08.017 23973415

[B36] Taupin P. (2007). BrdU immunohistochemistry for studying adult neurogenesis: paradigms, pitfalls, limitations, and validation. Brain Res. Rev. 53, 198–214. 10.1016/j.brainresrev.2006.08.002 17020783

[B37] Toner C. K. Pirogovsky E. Kirwan C. B. Gilbert P. E. (2009). Visual object pattern separation deficits in nondemented older adults. Learn. Mem. 16, 338–342. 10.1101/lm.1315109 19403797

[B38] Tronel S. Belnoue L. Grosjean N. Revest J. M. Piazza P. V. Koehl M.. (2012). Adult-born neurons are necessary for extended contextual discrimination. Hippocampus 22, 292–298. 10.1002/hipo.20895 21049483

[B39] van Praag H. Shubert T. Zhao C. Gage F. H. (2005). Exercise enhances learning and hippocampal neurogenesis in aged mice. J. Neurosci. 25, 8680–8685. 10.1523/JNEUROSCI.1731-05.2005 16177036 PMC1360197

[B40] Vivar C. Potter M. C. van Praag H. (2013). All about running: synaptic plasticity, growth factors and adult hippocampal neurogenesis. Curr. Top. Behav. Neurosci. 15, 189–210. 10.1007/7854_2012_220 22847651 PMC4565722

[B41] Voss M. W. Vivar C. Kramer A. F. van Praag H. (2013). Bridging animal and human models of exercise-induced brain plasticity. Trends Cogn. Sci. 17, 525–544. 10.1016/j.tics.2013.08.001 24029446 PMC4565723

[B42] Wang J. W. David D. J. Monckton J. E. Battaglia F. Hen R. (2008). Chronic fluoxetine stimulates maturation and synaptic plasticity of adult-born hippocampal granule cells. J. Neurosci. 28, 1374–1384. 10.1523/JNEUROSCI.3632-07.2008 18256257 PMC6671574

[B43] Wong-Goodrich S. J. Pfau M. L. Flores C. T. Fraser J. A. Williams C. L. Jones L. W. (2010). Voluntary running prevents progressive memory decline and increases adult hippocampal neurogenesis and growth factor expression after whole-brain irradiation. Cancer Res. 70, 9329–9338. 10.1158/0008-5472.CAN-10-1854 20884629 PMC2982943

[B44] Wu M. V. Hen R. (2014). Functional dissociation of adult-born neurons along the dorsoventral axis of the dentate gyrus. Hippocampus 24, 751–761. 10.1002/hipo.22265 24550158 PMC4222246

[B45] Yassa M. A. Lacy J. W. Stark S. M. Albert M. S. Gallagher M. Stark C. E. (2011). Pattern separation deficits associated with increased hippocampal CA3 and dentate gyrus activity in nondemented older adults. Hippocampus 21, 968–979. 10.1002/hipo.20808 20865732 PMC3010452

